# Embedding Consumer‐Led Research in a Comprehensive Cancer Centre Alliance: A Mixed‐Methods Evaluation

**DOI:** 10.1111/hex.70859

**Published:** 2026-09-08

**Authors:** Lucy Boyd, Claire Nightingale, Brad Astbury, Natalie Diepenhorst, Leslie Leckie, Michelle Barrett, Joanne M. Britto, Simonne Neil, Tessa Saunders

**Affiliations:** ^1^ Evaluation and Implementation Science Unit, Centre for Health Policy, Melbourne School of Population and Global Health University of Melbourne Melbourne Victoria Australia; ^2^ Victorian Comprehensive Cancer Centre Alliance Melbourne Victoria Australia

**Keywords:** cancer research, consumer, consumer‐led, evaluation, lived experience leadership, patient and public involvement, research prioritisation

## Abstract

**Introduction:**

As consumer involvement becomes increasingly recognised as an important part of health and medical research, new ways to involve consumers are actively being explored. This evaluation aimed to investigate the experience of consumers and other stakeholders involved in two pilot consumer‐led research projects within a comprehensive cancer alliance.

**Methods:**

The evaluation consisted of program document review, an online survey and individual interviews to explore the experience of a range of stakeholders. Analysis was via a convergent mixed methods approach.

**Results:**

Overall, 12 consumer participants and 11 other non‐consumer stakeholder participants took part in the evaluation. An in‐depth narrative of participants’ experiences was developed, from which five key components of effective consumer‐led research were identified: (1) Organisational commitment to consumer involvement at all levels, (2) appropriate time and resources, (3) training and education, (4) clearly defined roles and responsibilities, and (5) an ecosystem that values individuals and creates a cohesive group dynamic. While the two pilot projects shared similarities, their iterative and sequential nature enabled learnings from the first to be applied in real time to the second. Overall, the experience of the programs was positive, with both consumers and other stakeholders rating the pilots as successful and stating they were highly likely to recommend the program to others.

**Conclusion:**

This evaluation illustrated that the implementation of a framework for consumer‐led research pilot projects was perceived positively and enhanced consumer leadership in the research process. Additionally, it identified areas where power and decision‐making could be more evenly shared, thereby enhancing consumer leadership in the research process.

**Patient or Public Contribution:**

People with lived experience of cancer (including service users and caregivers) were involved in this evaluation in two ways. First, two consumers were part of the Victorian Comprehensive Cancer Centre Alliance evaluation team (L.L. and N.D.) and helped design the evaluation plan and data collection tools and reviewed findings and draft reports. N.D. contributed to the manuscript write‐up. Second, consumers who were part of the two pilot projects and had roles in consumer‐led research projects (*n* = 17) were invited to participate in the evaluation activities (online survey and/or interview) to share their experience. These consumers were also invited to an online feedback session with the evaluation team to sense‐check and confirm the evaluation findings prior to finalisation.

## 1 Introduction

Consumer involvement in health settings has significantly evolved over the past few decades and is now considered a crucial component of health and medical research [1, 2]. Various terminology is used to describe the involvement of, or engagement with, individuals and groups who have experience of a particular health issue or health service [3, 4, 5, 6, 7, 8]. In Australia, the commonly used term is ‘consumer and community involvement (CCI)’ whereas the United Kingdom, the United States and Canada use the term ‘patient and public involvement’ (PPI) [3, 4, 5, 6, 7]. Consumers can be patients, caregivers, family members, prospective or past service users or members of the public. Globally, researchers, funding bodies, academic journals and decision‐makers are increasingly using, encouraging and, in some cases, mandating the integration of lived experience through consumer involvement [1, 2, 9].

The growing recognition of consumer involvement in research reflects multiple benefits highlighted in recent systematic reviews [10, 11, 12]. Consumers have a legitimate expectation to be meaningfully involved in research that influences their health and care. Correspondingly, researchers have an ethical responsibility to ensure such involvement, particularly in publicly funded studies and in research concerning marginalised or seldom‐heard populations [8, 10]. Meaningful involvement strengthens the quality, relevance and impact of research by integrating real‐world insights and lived experience into study design, conduct and interpretation [8, 10, 13]. Evidence [10, 11, 13] indicates that consumer engagement improves research dissemination, translation into practice, and alignment of care and outcomes with patient preferences. It can also strengthen consent and recruitment processes, increase inclusion of minority groups, and generate richer findings [8, 11]. Moreover, consumer and public participation promotes transparency and accountability, extending research beyond academic and other institutional settings, thereby increasing public trust [8, 10, 11]. Underpinning consumer involvement is an ‘ethic of responsibility’ which entails a commitment to attend to diverse perspectives, foster trust, and enable two‐way learning between researchers and consumers [8, 14].

Recent systematic reviews [3, 6, 7, 12, 15] illustrate the progress made in involving consumers in research. However, there remains a paucity of research that is truly led by consumers. For instance, Peters and colleagues conducted a systematic overview of reviews and developed a framework for the evaluation of research co‐design in health. In this paper [16] they reported on 51 reviews and found that only six studies met their criteria for end‐user‐led research. One of the published examples [17] of consumer‐led research consisted of a mixed‐methods evaluation of a community‐based cancer support service, where consumers played active roles throughout the project. Consumers in this study chaired the steering committee, contributed to developing an overall research plan, helped develop survey and interview questions, interpret data and contributed to publication and dissemination of findings [17].

More broadly, consumer involvement in research presents several challenges. One concern is the risk of superficial or ‘tokenistic’ involvement where engagement is treated as a tick‐box exercise rather than a meaningful collaboration [6, 18]. Meaningful engagement requires time, elevated levels of resources and an understanding of engagement practices to implement properly [6, 18]. These demands may not align with research timelines, skills and capacities of research teams and budgets required to support involvement [6, 19]. Limited or superficial involvement can also arise when there is insufficient organisational readiness (strategy, structure, support) to involve consumers in a meaningful and effective manner. Additionally, power imbalance between consumers and research teams can limit the influence of consumer contributions [6, 20].

Approaches to consumer involvement are evolving to include co‐design, co‐production, research partnerships and consumer‐led research. While there is no single agreed definition of these approaches to engagement, co‐design is generally understood to refer to collaboration between a range of stakeholders, including service users and providers, in *designing* solutions to an identified problem and ‘co‐production’ relates to the *delivery and implementation* of solutions, again via collaboration between stakeholders including consumers [4]. Cancer Australia [2] describes consumer‐led research as the highest level of engagement, where consumers set priorities, lead major activities, and hold the balance of power, distinguishing it from co‐design, co‐production and co‐creation, where decision‐making is a partnership between consumers and researchers. Related terms such as ‘patient‐led’, ‘public‐led’, ‘community‐driven’ are sometimes used interchangeably with consumer‐led [3, 4, 5, 6, 7].

This ambiguity in terminology makes it difficult to navigate and interpret the literature on consumer involvement in research [5]. Many systematic reviews on consumer involvement or co‐design exist, however, they do not always differentiate between the varying levels of involvement, nor do they align their definitions across contexts. Some reviews have made this distinction and clearly outlined definitions of the different types of involvement [16].

There are limited examples [21, 22] of where consumer leadership has been integrated, at an organisational level, rather than individual, team or unit level, into research programs. By ‘organisational level’ we mean that consumer involvement is supported by the Board and organisational leadership and embedded within organisational structures, policies, funding mechanisms and accountability processes. One example [22], from Australia, found their program to be meaningful and useful, and identified key enablers such as resourcing, organisational support and researcher training. Other institutions may be striving for integration of consumer leadership at an organisational level, but very little published literature exists [20], possibly due to challenges in reporting this type of initiative.

### 1.1 Study Context and Aims

The Victorian Comprehensive Cancer Centre (VCCC) Alliance is Australia's leading comprehensive cancer centre providing integrated, multi‐site and multidisciplinary research, education and care to improve outcomes for people affected by cancer. The organisation has an intentional commitment to strengthening consumer involvement not only in research projects but across all levels of the organisation [23]. The VCCC Alliance operationalised *Cancer Australia's National Framework for Consumer Involvement in Cancer Control* through a strategy, including resourcing and consumer remuneration for integrating the expertise of lived experience in research and advocacy. Within this engagement activities were categorised as informing, consulting, involving, partnership and consumer‐led initiatives [2, 23].

As part of the implementation of this strategy, the VCCC Alliance committed to implement two consumer‐led pilot projects (see Box 1), embedded into two Programs within a Strategic Program Plan (2021–2024) [24]): the Personalised Cancer Care Program, which focused on personalised genomic testing and understanding the patient experience of accessing and receiving tumour genetic profiling, and the Value‐based Cancer Care Program, which was designed to contribute to the evidence base for implementing value‐based care, with a consumer‐led research study included to explore the values of Arabic‐speaking cancer patients utilising interpreter services. These pilots aimed to position consumers at the highest of the five levels of engagement outlined above: ‘Consumer‐led’. To support these pilot projects, a comprehensive consumer education curriculum was developed and implemented to equip consumers with subject knowledge and enhanced confidence. The implementation of the educational curriculum was conducted prior to the commencement of the actual research prioritisation and project execution phases.

Box 1 **About consumer‐led research pilot projects at the Victorian Comprehensive Cancer Centre (VCCC) Alliance**

**What is the VCCC Alliance?**

- The VCCC Alliance is in Melbourne, Victoria. It is an innovative collaboration of 12 member institutions combining expertise in cancer care, education, advocacy and research.
- This venture has diverse funding and support sources including Victorian and Commonwealth government, member organisation contributions, grants and philanthropic donations.

**How did the consumer‐led research projects come about?**

- In 2019, The VCCC Alliance developed a model for consumer engagement based on Cancer Australia's framework and used it to guide engagement practices.
- Consumer‐led is when the balance of decision‐making lies in the consumer perspective. For research, this means to set a research direction that directly aligns with consumers’ needs and values.
- The VCCC Alliance Strategic Program Plan (SPP) 2021–2024 [24] was developed with input from consumers and included 10 programs of work and over 40 individual projects. It piloted consumer‐led research within 2 programs.

**What were the consumer‐led research pilot projects?**

- There were two consumer groups established, one in Personalised Cancer Care Program (which focused on personalised genomic testing and understanding the patient experience of accessing and receiving tumour genetic profiling), and the other in Value‐based Cancer Care Program (designed to contribute to the evidence base for implementing value‐based care, with a consumer‐led research study included to explore the values of Arabic‐speaking cancer patients utilising interpreter services). Both groups were led by two consumer co‐chairs, and each group was supported by a Program Manager.
- Consumer groups were tasked with prioritising a research direction for a project within that program and working together with a researcher for project execution.
- The first pilot project started in 2020. It focused on the Personalised cancer care research area and was led by eight consumers, two researchers and five program staff. The second program started in 2021 consisting of nine consumers, two researchers and five program staff.
  More information including a timeline of the pilot projects can be found in Supplementary material.

Through this program, the VCCC Alliance aimed to further develop research capacity in consumers. They also aimed to provide implementation guidance which could be harnessed by other organisations.

**Figure 1:**
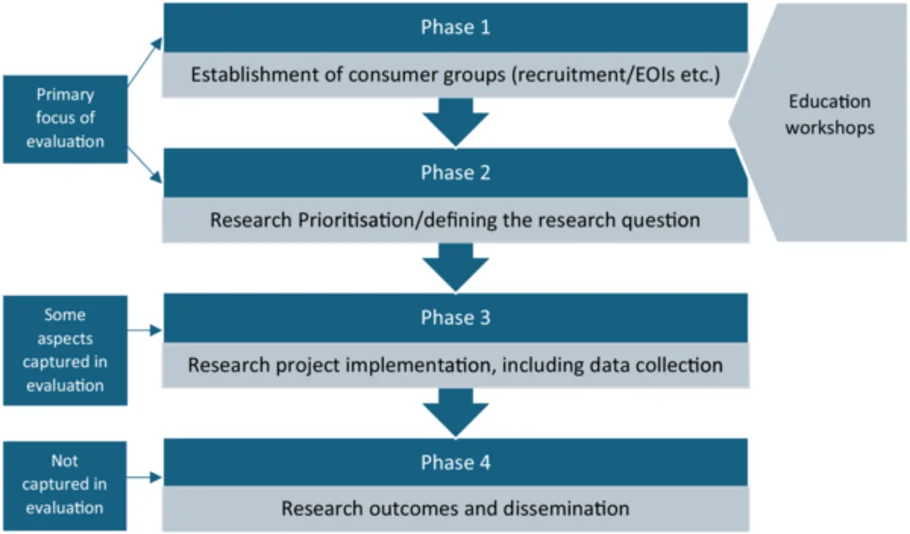
Pilot project phases included in evaluation.

This paper presents findings from an evaluation primarily of the first two phases of the consumer‐led research pilot projects at the VCCC Alliance. The first phase was the establishment of the consumer groups including expressions of interest and recruitment of consumer co‐chairs. The second phase was research prioritisation as a consumer group in each pilot project. Iterative steps were taken to define the research question and plan the research activities. Structured education tailored to the research focus was delivered throughout phase one and two. The third phase was the research project implementation including research activities such as data collection. Due to the timing of the evaluation, some aspects of the third phase were also captured in the evaluation but were not a focus of the evaluation. See Figure 1 below.

Overall, the evaluation explored:

i. Consumers and researchers, program and executive staff and other stakeholder experiences with the consumer‐led research pilot projects
ii. Barriers and enablers to implementing consumer‐led research.

Through this exploration, we aimed to identify the key components required to conduct consumer‐led research, which could inform implementation guidance for other organisations.

## 2 Materials and Methods

The research prioritisation activities were conducted from late 2021 to mid‐2023, with the evaluation conducted in the second half of 2024. The evaluation team comprised of four academics specialising in evaluation and implementation research (L.B., C.N., B.A. and T.S.). An evaluation advisory group comprising of the three staff spearheading the consumer‐led research program from VCCC Alliance (J.B., M.B. and S.N.) and two consumers with lived experience of cancer (L.L. and N.D.) guided the evaluation. The term ‘participants’ is used in this paper to refer to evaluation participants including consumers and other stakeholders who completed evaluation activities.

### 2.1 Evaluation Design

A convergent mixed methods design was employed [25, 26], with the primary purpose of using quantitative and qualitative data to achieve triangulation and complementarity in the evaluation across the two pilot projects [27]. A logic model (see Supplementary material S1) informed by the document review was first developed by the evaluation team and endorsed by the advisory group. Following this, an online survey and semi‐structured interviews with consumer participants (henceforth referred to as ‘consumers’), and program and executive staff, researchers and contractors supporting the two consumer‐led pilot projects (henceforth referred to as ‘other stakeholders’). This approach was chosen to enable the simultaneous collection and analysis of quantitative survey data and qualitative interview data, allowing for systematic comparison around a shared program logic model, while also accommodating the evaluation timeline.

The key components of consumer‐led research were identified through this systematic comparison of survey, interviews, documentary review and literature data, and by iterative sense‐making and validation with consumers and other stakeholders.

### 2.2 Survey and Interview Guide Development

The survey items and interview guide were purpose‐built for the project. Survey items and interview guide were developed iteratively through consultation with the evaluation advisory group to ensure relevance and appropriateness. The survey, housed in REDCap, comprised two sections, the first of which was identifiable and collected demographic information. The second section remained anonymous and included items about participant experience of the program, perceptions of the program purpose and importance, how they felt people worked together in the program, how likely they were to recommend it to others and how successful they felt it was. To ensure participants felt comfortable sharing openly, sections were split to maintain anonymity due to close working relationships and potential power imbalances allowing participants to respond candidly about their experiences without the risk of identification. Both sections used Likert scales, question matrices and open‐ended free text responses to elicit data. Participants provided informed consent through REDCap's e‐consent process for the survey and/or interview. See supplementary material S1 for copy of data collection tools.

The semi‐structured interview guide comprised several open‐ended questions regarding participant perception of consumer involvement and consumers leading research, of the program itself and of any challenges faced throughout the process. See Supplementary material S1 for example guide.

### 2.3 Data Collection and Recruitment

All consumers and other stakeholders involved in the pilot programs were invited to participate in the survey and an interview via an email from the Senior Manager of Consumer Involvement (J.B.). The invitation email contained a link to the REDCap form which included study information, consent form and contact details for the evaluation team. Consumer participants were offered remuneration in accordance with an existing VCCC Alliance cost schedule for consumer involvement [23]. Other stakeholders were not provided remuneration for their participation as the evaluation was regarded as a core activity of their paid work.

Interviews were conducted using videoconferencing by the evaluation team (L.B., C.N. and T.S.). Interviewers completed a ‘debrief report’ directly after the interview to capture key concepts from the discussion. Interview recordings were transcribed verbatim (Otter.ai) and checked for accuracy by the research team. Participants were given the opportunity to review and edit these prior to analysis. Eleven participants opted to review their transcripts, of which four made minor changes prior to analysis.

### 2.4 Analysis and Integration

Consumer participants were disaggregated from other stakeholders for analysis. Quantitative survey data were analysed in SPSS software to produce descriptive statistics and summaries of responses.

Simultaneously, qualitative data including interview transcripts, debrief reports and long responses from the online survey were analysed inductively and thematically by three authors (L.B., C.N. and T.S.) guided by the Framework Method which is used extensively in health services research to operationalise qualitative analysis [28]. This method consisted of transcription, familiarisation and initial coding, themes were developed and a draft matrix or, working analytical framework, of key components for establishing consumer‐led research was developed. This was reviewed and discussed by the evaluation team through iterative analysis until no new themes emerged and saturation and agreement were reached. The analytical framework was then applied, and remaining data were charted into the framework matrix ready for interpretation and integration with the quantitative data. Alongside the key components, the analysis also produced several narratives of participant experiences from a range of perspectives in the two consumer‐led pilot projects.

The qualitative and quantitative analyses were brought together to compare and relate findings [25, 26]. Following the principles and practices outlined by Fetters, Curry and Creswell [29], all data were interpreted concurrently, and descriptive statistics were mapped alongside the key components for the establishment of consumer‐led research and narratives of participants’ experiences. [29] Throughout this process, the evaluation team frequently met and discussed findings and interpretation. Findings were also discussed with the evaluation advisory group for further refinement.

All consumers invited to participate in the evaluation were invited to a subsequent 1‐h online workshop to review and sense‐check the five components. Consumers involved in the pilot programs could attend the workshop regardless of their participation status in the survey and/or interview; attendance was voluntary, and consent was implied. Notes were taken during the workshop, but no recordings were obtained. Adjustments to the draft evaluation findings including the five components were made, particularly around word choice and emphasis on the significance of some key components before final interpretation.

Triangulation was used to cross‐validate findings across the same evaluation questions and logic domains and complementarity added depth by situating survey patterns within participant narratives. Integration was intentional and dynamic, occurring across three key phases: design, analysis, and interpretation, consistent with Bazeley's [30] conceptualisation of mixed methods integration as an iterative, dynamic process. Where findings diverged, greater interpretive weight was afforded to the qualitative strand because it better captured contextual conditions, rationales and lived experience [31].

#### 2.4.1 Ethical Approval and Considerations

Ethical approval was sought, and approval received on 20/05/2024 from the University of Melbourne Human Research Ethics Committee (Project ID: 29626).

To overcome potential bias and to reduce possibility of re‐identification, one section of the survey was collected anonymously and stored in a separate database to demographics and potentially identifiable information.

## 3 Results

### 3.1 Participants

All 34 individuals involved with the pilot projects (17 consumers and 17 other stakeholders) were invited to take part in the evaluation. Twenty‐three individuals (12 consumers and 11 other stakeholders) completed at least one evaluation component (response rate 68%). Twenty completed the survey and 17 participated in an interview (Table 1).

**Table 1:** Participant characteristics and evaluation component completion.

Participant characteristics	Consumer participant	Other stakeholders	Total sample
Evaluation component	12	11	23
Completed both survey and interview	6	8	14
Completed survey only	4	2	6
Completed interview only	2	1	3

Of the 20 survey respondents most (*n* = 16, 80%) were female. The mean age of consumer respondents was 53.6 years, and the mean age of other stakeholders was 44.3 years. Eight participants reported being born overseas. Three participants (two consumers) reported speaking a language other than English at home. Most consumer participants (*n* = 7, 70%) had experience as a carer or support person for someone with cancer and about half (*n* = 4, 40%) said they had experience as a person with cancer, one person declined to answer. Almost all consumer participants (*n* = 9, 90%) indicated they had some previous experience as a consumer representative or advocate. Due to small cell counts, these data have not been displayed in table format. Further, due to ethical considerations, including the need to protect privacy, maintain consent standards and avoid bias or assumptions, this study did not collect any data on individuals who did not provide consent or participate in evaluation activities.

Four other stakeholder participants reported prior completion of training or education on consumer involvement in research in other professional contexts (*n* = 4, 40%). These four, plus an additional three (*n* = 7, 70%), had worked with consumers before and described a breadth of experience in this space including research projects and clinical work, co‐authoring publications, utilising consumers as educators and working with advocacy groups across both cancer and other health conditions and a range of organisations.

### 3.2 Perceptions of Success

Participants frequently mentioned high levels of satisfaction with the pilot projects, positively reflecting on organisational factors such as existing consumer engagement and a culture that values consumers. In an interview, one consumer (interview ID17) commented ‘I think it went really smoothly. I think the researcher and the program manager did a really wonderful job’. Similarly, one of the other stakeholder participants (interview ID15) reflected they were ‘very supportive of the overall idea. Excited about it. (…) It [consumer leadership] makes the work more enjoyable as a researcher, more energising’. The survey results supported this qualitative finding, with an average success rating of 8.1/10 from consumer participants and 7.5/10 from other stakeholder participants. Similarly, both groups were highly likely to recommend a similar program to others.

However, many discussed uncertainties about how to define success for the pilot projects—whether this should be measured via process or outcomes, and this varied between consumers and other stakeholders due to this not being defined at the start:

The success was in the process and what others can learn about high levels of consumer engagement and its benefits.

(Consumer, survey ID17)

Unfortunately, I have not really seen any results from the program, so it is quite hard for me to judge the success. For me, I think success would be: 1. Development of a consumer‐led program 2. Completion of a consumer‐led research project 3. Publication of consumer‐led research project.

(Other stakeholder, interview ID20)

The timing of the evaluation and its focus on the establishment and prioritisation phases of the consumer‐led projects meant that it took place prior to the production of research outputs, which may have contributed to the varied perceptions of what success meant in this context.

Both consumer and other stakeholder participants identified flaws or challenges faced by the pilot projects. These included feasibility difficulties, challenges related to novel approaches or populations and misalignment of the research topic with existing infrastructure and projects. Consumers and other stakeholders suggested ways to improve this:

So they've [the consumer group] designed their own question that doesn't relate to anything else. It would have been much easier, and probably more impactful, if they had worked with one of the existing researchers on one of the projects and designed a question with them that complemented research that was already underway.

(Other stakeholder, interview ID04)

### 3.3 Key Components

Five key components essential to the establishment of consumer‐led research within an organisation were identified from our analysis and interpretation of the data, they were refined and ‘validated’ by input from stakeholders, including consumers. The key components are: (1) Organisational commitment to consumer involvement at all levels, (2) appropriate time and resources, (3) training and education that meet the needs of participants and program, (4) clearly defined roles and responsibilities that align with consumer‐led research, and (5) an ecosystem that values individuals and creates a cohesive group dynamic.

#### 3.3.1 Key Component 1—Organisational Commitment to Consumer Involvement at All Levels

All participants identified that organisational commitment to consumer‐led research was valuable, and that this was something the VCCC Alliance had succeeded in. This strong commitment was seen to facilitate: (i) a supportive culture that welcomes and values the contributions of consumers, (ii) high levels of motivation among consumers and non‐consumer stakeholders, (iii) organisational readiness, and (iv) a commitment to learn and improve. In an interview, a consumer (interview ID08) stated ‘The great benefit of the VCCC Alliance doing it was that they really were committed to the idea. And it's a bold move to cede control to a group of consumers and in general, they stuck with it’.

A key example of supportive organisational readiness was the structures in place to support consumers—including having existing consumer relationships, having consumer involvement as part of the organisational strategy, having a remuneration policy, and documented processes and practices for engagement. Confirming this, one of the other stakeholders stated *‘*…to conduct something like this, executive support is instrumental…it wouldn't have happened without executive backing’ (interview ID06). Participants reflected that having existing consumer representatives on steering committees and their role outlined in the strategic plan was greatly beneficial as this fostered accountability. Another supportive feature was that the VCCC Alliance had a dedicated staff member, with experience in PPI in research to manage consumer involvement.

The survey included a matrix asking consumers to rate statements (not at all, slightly, moderately, very or extremely) about their time working with others during the pilot projects. There were seven statements in total including ‘how much did you feel that your contributions were valued?’, ‘how much did you feel included in conversations/decisions?’. All seven statements had > 80% of respondents selecting ‘very’ or ‘extremely’. One to two participants rated five of the seven statements ‘moderately’. The option ‘slightly’ was chosen once and this was for the statement ‘when working with others in the consumer‐led research, how much did you feel you were supported to contact other team members including other consumers, facilitators *and* researchers?’. These survey results indicate the extent to which consumers felt their feedback was heard and included was another success of the pilots, reflective of an organisational willingness to learn and adapt, and of an organisational culture that embraces and values consumer voices.

In a matrix of statements about working together, all items received ratings of at least 80% positive responses (‘very’ or ‘extremely’).

#### 3.3.2 Key Component 2—Appropriate Time and Resources

Central to the key component of ‘appropriate time and resources’ were: (i) consumer time and remuneration, (ii) other stakeholder time and resources and (iii) managing expectations.

One of the biggest challenges identified by most participants was having enough time and resources to support the consumer‐led research. As this was a new endeavour, participants acknowledged this as a risk, and the VCCC Alliance team attempted to budget appropriately for the time and resourcing required.

Many participants, including consumers, program staff and researchers, felt they contributed more than they had expected, and for some, more than they had been remunerated for:

The collaborative agreement that we put in place just didn't have enough hours associated with it. And I totally understand, for the researcher, this isn't [the] only work they do, and they have to be really mindful about how much time they associated things and how they're being appropriately remunerated.

(Other stakeholder, interview ID07)

The sub‐component of ‘managing expectations’ centred around what can be achieved in a research project with the available resources. One researcher noted that they were committed to delivering something that aligned with consumer expectations but were restricted by resources and felt that this disappointed the consumers: ‘putting in some boundaries for my own time because the proposal that I put together for the budget that they had available, I've probably spent more hours on the project than that’ (Other stakeholder, interview ID15). These expectations were taken seriously by researchers, who appeared to absorb this responsibility, and felt frustration directed at them.

Staff turnover was a key factor that affected the progress of the pilot projects, creating time and resource challenges that left some consumers feeling disappointed and disheartened by the research process.

#### 3.3.3 Key Component 3—Training and Education That Meets the Needs of Participants and Program

Training and education were viewed as critical to the program's success with most participants reflecting positively on this. This component highlights: (i) the importance of building consumers’ confidence and competence to contribute meaningfully to research prioritisation, alongside enhancing researchers’ capacity to work effectively with consumers, and (ii) proposed improvements to strengthen education and training.

Many participants, both consumers and other stakeholders, reflected on how much they had learned throughout the project, not only through formal training and education but also through engagement in the process itself. One participant highlighted that being provided with sufficient background information and context enabled meaningful engagement and supported their ability to contribute to formulating a research question:

I think they did really well at kind of equipping us with enough information about research and context and other sort of relevant research areas that have already been done previously in the things that have value‐based cancer care realm. They did very well with that, and that I felt like I had enough tools to sort of be able to formulate the research question with the group, I think they did a really, really fantastic job at that.

(Consumer, interview ID17)

Having both subject matter expertise and relevant lived experience was considered crucial for prioritising research questions. Some participants felt that further training on its importance and more time to scope the project's relevance would be beneficial:

We should have spent more time around that initial preparatory work because the group was inexperienced in research, just because I know that there's a need out there as a consumer or a patient does not automatically translate into a research question.

(Consumer, interview ID12)

Most consumers reflected positively on the education and agreed that the education covered information required for the program.

Consumers indicated their confidence and competence as consumer leaders in research had grown. This was reflected in the high proportion of consumers who said they intended to apply the knowledge, skills, and attitudes they learned in this program as a cancer consumer, with 80% of survey respondents strongly agreeing with this statement. Other stakeholders also reported increased knowledge and confidence in their roles working with consumers. Suggested improvements to the education and training included having a less didactic style for some of the content delivery and stronger reiteration of earlier learnings at relevant times throughout the program instead of at the start.

#### 3.3.4 Key Component 4—Clearly Defined Roles and Responsibilities That Align With Consumer‐Led Research

Establishing and communicating clear roles and responsibilities was a central topic in interviews with most participants. Consumers and other stakeholders felt that the pilot projects had provided important learnings about roles and responsibilities not only for the VCCC Alliance going forward, but also for other organisations embarking on a similar endeavour. This key component focuses on three factors: (i) establishing and communicating roles and responsibilities (via a Responsible, Accountable, Consulted and Informed [RACI] matrix, see Box 2), (ii) responsibility, accountability and integrity of consumer‐led research and (iii) limitations or challenges of roles.

Box 2 **RACI Matrix**

**R: Responsible.** Need to have at least one of these per task. They are responsible for the correct completion of the task.

**A: Accountable**. Need to have at least one of these per task. This is the role that are ultimately held accountable, they need to sign off on decisions and work by those who are Responsible.

**C: Consulted.** Opinions are sought, two‐way communication.

**I: Informed.** They are kept up‐to‐date on progress, usually at the completion of a task or reaching a milestone. One‐way communication.

Participants reflected that the successful use of a RACI matrix, as a tool and part of the process to define roles and responsibilities, was a key learning to share with others. This tool shows, for each stage of the project, whether someone should be ‘Responsible’, ‘Accountable’, ‘Consulted’ or ‘Informed’. Categorising roles and tasks in the matrix helped to clearly define everyone's obligations and level of involvement at each stage.

At the outset of the pilot projects, further clarification was requested from consumers and other stakeholders to confirm what consumer‐led research meant, with few confident in their understanding at commencement. However, many participants stated they experienced an evolution or development of the definition and understanding of consumer‐led research throughout the pilot projects. It was perceived to be ‘a welcomed change in understanding, initially thought we would just be approving decisions already made but we had thorough involvement, from start to finish with key decision‐making being made by consumers, actually leading the project’ (Consumer, survey ID15).

Many participants reflected on the challenge of allowing consumers to have the decision‐making power, and the implications that this had for governance, and accountability, particularly when consumers are not employees of the sponsor organisation:

And it's very difficult for someone like VCCC Alliance to say ‘well this is consumer‐led, they make all the key decisions’. The reality is that we're not staff members. And eventually, staff members want to take accountability for various things. And that's probably the challenge of the consumer‐led research now and into the future, in how much responsibility and accountability to cede to the consumer‐led group with confidence.

(Consumer, interview ID08)

Some consumer participants felt limited in their roles as consumers, acknowledging that while their lived experience with cancer was valued, their broader professional and personal skills were not necessarily acknowledged. This sentiment was also acknowledged by other stakeholders, who reflected that it was not necessarily ethical to take advantage of consumer professional skills.

Yes, bring your whole self, bring your ideas, but it's actually unethical for us to you know, if you're a graphic designer, ask you to design our pamphlet ‐ we haven't paid you for that. And we haven't gone to market on that.

(Other stakeholder, interview ID04)

The survey asked respondents to reflect on the opportunity the program provided for different levels of involvement. The majority agreed that they had been given the opportunity to provide strategic advice on consumer‐led education and research and thought that their engagement with governance, strategy, policy and evaluation were sufficient.

#### 3.3.5 Key Component 5—an Ecosystem That Values Individuals and Creates a Cohesive Group Dynamic

All participants, both in the survey and interviews, emphasised the importance of people and the group dynamic. Consumer participants widely agreed that working together as a group offered a better experience than working as individual consumers on a project, and they considered this a key strength. Participants also commonly noted the challenge of managing the power dynamic between researchers, consumers and management teams. When reflecting on their group experience, they highlighted: (i) the value of the group as a whole, (ii) the diversity, dynamics and ‘fit’ of the group, and (iii) the need to balance power while maintaining momentum.

Consumer participants from both pilot projects felt they worked well as a group, collectively deciding how they would work together, so that everyone felt they made a valuable contribution. The role of program managers, researchers and other facilitators in supporting groups was also seen as a factor that contributed to this success, as was the partnership between the consumers, and the researcher. In an interview, a consumer stated ‘our researcher that we chose was extremely good. An incredibly good communicator. [And], a proficient researcher as well’ (Consumer, interview ID02).

It is important to note that research teams traditionally develop organically over time, building relationships, work plans, and iterative processes responsive to needs and opportunities. This contrasts with how consumer‐led research was piloted at the VCCC Alliance, where participants were brought together specifically for the project rather than building on an established team. Although most consumers had pre‐existing relationships with the organisation, the lived experience of individual group members did not always match the specific focus of the project, for example, one project had a focus on Arabic speakers but members of the consumer group did not have this background.

Similarly, relationships with researchers were often new, and participants may not have been working in areas of personal passion. In this sense, some were ‘artificially’ grouped based on their status as consumers rather than on aligned lived experience, which presents unique challenges for fostering cohesion and meaningful contribution.

The group's diversity was also seen as a strength by the consumers in the group, with a consumer commenting ‘I would certainly recommend the richness of diversity. And that was really, really excellent’ (Consumer, interview ID02). The group dynamic was appreciated by consumer participants and is reflected in the predominantly positive responses in the survey as mentioned in Key Component 1.

## 4 Discussion

This evaluation aimed to investigate the experiences of participants in two pilot projects designed to provide consumers with a leadership role in research prioritisation [17, 32, 33, 34]. The present consumer‐led projects were similar in approach to the few published examples about this growing area, and experienced challenges similar to others described in Australian and international literature [17, 32, 33, 34, 35, 36]. Our findings, which align with the underlying logic model (see Supplementary material S1), confirm that this approach was broadly acceptable to all involved and highlight five key components outlining what is required to implement these initiatives. Further, we highlighted enablers and barriers within each of these components that organisations should consider when adopting a consumer‐led approach.

Our evaluation revealed that participants’ understanding of consumer‐led research was heterogeneous and developed throughout the project. This was evident in reported shifts in understanding throughout the pilot program and in the varied definitions provided by consumers and other stakeholders. The consequences of this included unclear ownership of outputs and unease when making decisions. These findings contributed to the identification of key component 4 (Clearly defined roles and responsibilities that align with consumer‐led research). Other studies have reported that this can be a challenge when involving consumers in research [15, 36, 37, 38, 39]. Unclear roles and responsibilities are often cited as sources of tension, which was also apparent in our evaluation. While efforts were made to clarify roles and responsibilities, for example using the RACI matrix, some uncertainty and confusion remained. For example, publicly funded organisations have a responsibility to funding bodies as well as their existing organisational vision and values. This responsibility may ultimately override decisions made by consumers leading a project. While these restrictions are necessary, they could dilute or impede the role of consumers as leaders in research [35]. Further research is needed to investigate effective strategies to address this issue.

Selecting consumers with diverse lived experiences and backgrounds, while still having lived experience aligned with the research topic, emerged as another challenge [39]. This was particularly evident in Key Component 5 (An ecosystem that values individuals and creates a cohesive group dynamic). While prior research presents mixed results on the value of consumer diversity, all studies acknowledge that achieving this is a challenge [19, 34, 36, 40]. In our study, participants reflected positively on the diversity of consumers, as a strength of the project, noting the inclusion of a range of ages and backgrounds. However, some consumers questioned whether the diversity was appropriate for the specific research topic. They argued that consumer selection should be aligned with the research focus, and that lived experience should be directly relevant to this. In the context of projects where the research focus is determined after consumer selection, this may require additional recruitment or engagement strategies to ensure relevant lived‐experience perspectives are incorporated.

As previous studies have found, consumer motivation and genuine interest were crucial for consumer involvement or consumer‐led research [17, 19, 34, 36, 40]. Schilstra and colleagues [19] identified pre‐established consumer networks as an enabler of consumer‐led research. Additionally, insights from the Women's Health Research, Translation and Impact Network (WHTRN) [21] further support these findings. Their consumer and community involvement strategy highlights the importance of early and broad engagement, meaningful partnerships and genuine contributions from consumers. The process used to develop their strategy provides a practical roadmap for other institutions seeking to embed consumer involvement throughout the research pathway [21]. Our findings are consistent with this and led to the identification of Key Component 1 (Organisational commitment to consumer involvement at all levels), with many consumers in our study highlighting that their existing relationship with the VCCC Alliance facilitated engagement.

Existing consumer involvement evaluation frameworks increasingly seek to assess the impact of consumer involvement either through a primary focus on research impact or through combined process and impact approaches [39, 41, 42, 43]. This reflects a broader shift in the field, driven in part by funder expectations for demonstrable impact and the growing demand for practical tools to capture and evidence the value of consumer involvement.

Our evaluation contributes to this developing literature by foregrounding the processes of consumer‐led research, particularly how consumers work with researchers and other stakeholders. While this places greater emphasis on process than on the measurement of downstream research impacts, these domains are closely interconnected. Indeed, our findings highlight the challenges participants experienced in defining and recognising ‘success’, underscoring the often indirect, diffuse, and negotiated nature of impact in consumer involvement.

In this sense, our approach complements existing impact‐focused frameworks by providing a more detailed account of the relational and organisational conditions through which impact is generated. Understanding how involvement is experienced, enacted, and supported is critical for interpreting observed impacts and for strengthening future practice. Rather than positioning process and impact as competing priorities, our findings suggest the need for integrated evaluation approaches that capture both the quality of consumer involvement processes and their contribution to meaningful research outcomes.

### 4.1 Strengths and Limitations

To our knowledge, this is the first mixed‐methods evaluation of consumer‐led research in a large comprehensive cancer centre. Strengths of the evaluation include the evaluation design, consumer representation on the evaluation advisory group, inclusion of both consumer and ‘other stakeholder’ perspectives, and a workshop with pilot project consumers to confirm findings.

The findings of our study should be considered with the following limitations in mind. Most, but not all potential participants (68%) completed the evaluation components. It is unclear why some potential participants did not complete the evaluation. As such, there may be self‐selection bias in those who completed it. This was further constrained by the small number of individuals involved in the pilot projects and ethical considerations to capture demographics or characteristics of non‐participants. Some logistical or timing limitations are that the evaluation was designed and conducted retrospectively. Ideally, this would occur simultaneously with the design of the project and throughout the various processes. Due to ethics and the fact this was a commissioned real‐world evaluation scenario rather than a research grant where this could be planned out, the timing of the evaluation did not align with the activities of interest. This was ~18 months after the research prioritisation phase (Phase 2) and some recall bias may be present. Additionally, the pilot projects were ongoing, so full outcomes had not been realised. Lastly, while the survey items were developed based on a review of literature and were refined by the VCCC Alliance evaluation advisory group, it was beyond the scope of the study, and the sample size was too small to assess the validity and reliability of the survey items. This evaluation was conducted at a single study site with a relatively small sample, which limits the generalisability and transferability of the findings. We note that previous research suggests heterogeneity of implementation of consumer involvement between countries so our findings may differ from experiences in other cultural settings or different contexts [44]. The transferability of findings may be limited as most consumer participants had prior experience as advocates or consumer representatives, which may have influenced their confidence, expectations and familiarity with research and research processes.

### 4.2 Implications for Future Programs and Research

The five key components identified in this evaluation provide a platform for other institutions and organisations to implement consumer‐led research and some guidance for them to build their own tailored models. The evaluation also presents insight into the positive experiences that both consumers and other stakeholders can gain from consumer‐led research.

Recommendations from this evaluation for other organisations or future research include the importance of having a focus on genuine connection and involvement. An emphasis on understanding your ‘why’ and going beyond tokenistic engagement is powerful to both consumers and other stakeholders. More research is needed regarding the funding and sustainability of these projects.

## 5 Conclusion

Overall, this evaluation showed that the implementation of the consumer‐led research framework for the consumer‐led pilot projects was seen to have contributed to a network of consumers with the competence and confidence to take on future roles and continue contributing to consumer‐led research and consumer involvement in research more broadly. The evaluation approach outlined here may offer a practical, adaptable way for researchers to capture consumer‐led research, addressing current demands from funders for demonstrable impact. Future research could explore a similar model in different settings or contexts, including beyond cancer research.

## Author Contributions

**Lucy Boyd:** conceptualisation, formal analysis, funding acquisition, investigation, methodology, project administration, visualisation, writing – original draft preparation, writing – review and editing. **Brad Astbury:** conceptualisation, formal analysis, funding acquisition, methodology, supervision, visualisation, writing – review and editing. **Claire Nightingale:** conceptualisation, formal analysis, funding acquisition, investigation, methodology, supervision, visualisation, writing – original draft preparation, writing – review and editing. **Natalie Diepenhorst (consumer representative):** conceptualisation, methodology, writing – review and editing. **Leslie Leckie (consumer representative):** conceptualisation, methodology. **Michelle Barrett:** conceptualisation, methodology, writing – review and editing. **Joanne Britto:** conceptualisation, methodology, writing – review and editing. **Simonne Neil:** conceptualisation, methodology, writing – review and editing. **Tessa Saunders:** conceptualisation, formal analysis, funding acquisition, investigation, methodology, supervision, visualisation, writing – review and editing.

The Victorian Comprehensive Cancer Centre Alliance is supported by the Victorian Government. L.B., T.S., B.A. and C.N. were contracted by the Victorian Comprehensive Cancer Centre Alliance to conduct this work and were supported by VCCC Alliance Strategic Program Plan funding through the Victorian Government. Open access publishing facilitated by The University of Melbourne, as part of the Wiley ‐ The University of Melbourne agreement via the Council of Australasian University Librarians.

## Ethics Statement

Ethical approval was sought, and approval received on 20 May 2024 from the University of Melbourne Human Research Ethics Committee (Project ID: 29626). Written informed consent was obtained for all survey and interview participants.

## Conflicts of Interest

M.B., J.B., S.N., N.D. and L.L. were participants in the study and/or employed by the Victorian Comprehensive Cancer Centre Alliance, which commissioned the evaluation. All authors report this as a potential competing interest. The potential conflict was managed through transparent reporting, independent analysis and oversight by authors not employed by the commissioning organisation (L.B., C.N., B.A. and T.S.).

## Supporting information


Supporting File

## Data Availability

The survey and interview data reported in this article are not publicly available to meet the terms of ethical approval for the study and to protect participant privacy.
