# Supplementary material for: Embedding Consumer‐Led Research in a Comprehensive Cancer Centre Alliance: A Mixed‐Methods Evaluation
Source: Health Expect. 2026 Sep 8;29(5):e70859. doi: 10.1111/hex.70859 (PMC13554987; doi:10.1111/hex.70859)
Supplement: Supplementary file 1 [file HEX-29-e70859-s001.docx]

Supplementary material

**Title:** Embedding consumer-led research in a comprehensive cancer centre alliance: a mixed-methods evaluation

**Journal:** Health Expectations

# 1 Logic model

# 2 Timeline

# 3 Questionnaire

**PART 1 – CONSUMER QUESTIONNAIRE**

Project: Evaluation of VCCC Alliance Consumer-led Research Program

<redacted logo>

<Department and school name - redacted>

***Ethics project ID:*** <redacted>

**Thank you for your interest in participating in this evaluation project. For more information about this project, please see attachment here <PDF attachment of PLS>**

**We are interested in your thoughts on consumer-led research and how well this is working in the VCCC Alliance program(s) you are involved with.**

**The following questions are divided into two parts:**

Part 1 - About you (includes identifying information to measure demographics etc. Responses will be de-identified for analysis and reporting)

Part 2 - About your experience and views (stored separately to Part 1, no identifying information)

**Together, they will take 10-15 minutes to complete.**

1. I consent to participate in this project, the details of which have been explained to me, and I have been provided with a written plain language statement **<linked to document>** to keep.
2. I understand that the purpose of this research is to evaluate the VCCC Alliance consumer-led research framework and associated pilot projects.
3. I understand that my participation in this project is for research/evaluation purposes only.
4. I acknowledge that the possible effects of participating in this research project have been explained to my satisfaction.
5. In this project I will be required to complete a questionnaire that will take approximately 15 minutes.
6. I understand that my participation is voluntary and that I am free to withdraw from this project anytime without explanation or prejudice and to withdraw any unprocessed data that I have provided.
7. I understand that the data from this research will be stored at the <redacted> and will be destroyed 5 years after publication.
8. I have been informed that the confidentiality of the information I provide will be safeguarded subject to any legal requirements; my data will be password protected and accessible only by the named researchers.
9. I understand that by ticking the following box I am agreeing to take part in this research.

- **Yes, I agree to take part in this questionnaire**
- **No, I do not agree to take part in this questionnaire (this option will exit the survey)**

For more information, please contact the evaluation team:
Responsible Researcher: <redacted>

Tel: <redacted> Email: <redacted>  

Additional Researchers:
<redacted> Email: <redacted>

<redacted> Email: <redacted>

<redacted> Email: <redacted>

| **Evaluation of VCCC Alliance Consumer-led Research Part 1** | A A A |
| --- | --- |

**Page 2 of 3**

| Part 1 - About you | | |
| --- | --- | --- |
| **First name:** |  |  |
| **Last name:** |  |  |

1. What was your role in the VCCC Alliance Consumer-led Research Program?
   a. Consumer co-chair
   b. Consumer member
   c. Education development and delivery
   d. VCCC Alliance executive
   e. Researcher
   f. Educator/facilitator
   g. Program manager
   h. Other consumer (please describe)
   i. Other non-consumer (please describe)
2. How do you describe your gender?

*Gender refers to current gender, which may be different to sex recorded at birth and may be different to what is indicated on legal documents. Please select one box:*

a. Man or male
b. Woman or female
c. Non-binary
d. I use a different term (please specify)
e. Prefer not to answer

3. What was your age last birthday?_____________
4. In which country were you born?
   a. 11. Australia
   b. 12. England
   c. 13. New Zealand
   d. 14. India
   e. 15. Philippines
   f. 16. Vietnam
   g. 17. Italy
   h. 18. South Africa
   i. 19. Malaysia
   j. 20. Scotland
   k. 99. Other
5. Are you of Aboriginal origin, Torres Strait Islander origin, or both?
   a. Aboriginal
   b. Torres Strait Islander
   c. Both Aboriginal and Torres Strait Islander
   d. Neither Aboriginal or Torres Strait Islander
   e. Prefer not to answer
6. Which language do you mainly speak at home? Note, if more than one language, indicate the one that is spoken most often.
   a. English
   b. Mandarin
   c. Arabic
   d. Cantonese
   e. Vietnamese
   f. Italian
   g. Greek
   h. Hindi
   i. Spanish
   j. Punjabi
   k. Other (please specify)
7. What is the level of the highest qualification that you have completed?
   a. Postgraduate Degree
   b. Graduate Diploma/Graduate Certificate
   c. Bachelor Degree
   d. Advanced Diploma/Diploma
   e. Certificate III/IV
   f. Year 12 or equivalent
   g. Year 11
   h. Year 10
   i. Below Year 10/No educational attainment
8. What is your home postcode? (_ _ _ _)
9. Which of the following best describes your current employment status?
   a. Full-time employee
   b. Part-time employee
   c. Self-employed – not employing others
   d. Self-employed – employing others
   e. Unemployed – seeking part- or full-time work
   f. Not employed – not seeking employment
   g. Other
10. The next question is interested in your experience as a consumer representative PRIOR to working with the VCCC Alliance on the consumer-led research pilots. This means any activity before <date of program>.

If you have experience in more than one of these, you can select multiple options.

“As a consumer I have:”

- reviewed grant applications
- reviewed ethics applications
- contributed to consultations (this could be a written submission or attending a meeting)
- been involved in governance
- completed surveys and interviews
- attended workshops and meetings
- other: please describe
- not had any formal/informal roles prior to this program (selecting this deselects all other options)

*<if other, free text to describe other involvement>*

11. as a consumer, are you..? (can select multiple)
    a. A person/patient with experience of cancer
    b. A carer or support person for someone with cancer
    c. Other (please describe)
12. If comfortable, please provide some more detail about your experience with cancer: (include type of cancer, stage at diagnosis, years since diagnosis etc) (free text)

**PART 2 – CONSUMER QUESTIONNAIRE**

**The remaining questions ask about your experience and views of the consumer-led research program. All answers beyond this point will be anonymous. They will not be connected to your previous answers.**

## 3.1 Perceived purpose, role and responsibility of consumers in research

1. To make sure we ask you the right questions, were you a consumer or other stakeholder in this program?
   a. I was/am a consumer in this program
   b. I was/am another stakeholder in this program (this may include educators, facilitators, VCCC Alliance executive staff, program managers, researchers etc.)
2. Which program of work are/were you involved in?
   a. Program 2: Personalised cancer care
   b. Program 5: Value-based cancer care
3. How did you first hear about this particular consumer opportunity? (free text)
4. What was it about this particular consumer activity that motivated you to apply? (free text)
5. In your opinion, what is the purpose of consumer involvement in research, in general? (free text)
6. In your own words, what was the main aim of the VCCC Alliance Consumer-led Research Program? (free text)

## 3.2 Definition of consumer-led research

7. The following activities define consumer-led engagement. Please rate your level of agreement with the following statements in the context of this program: *(select one option for each row)*

*"As a consumer in this program I..."*

|  | Strongly disagree | Disagree | Neither agree nor disagree | Agree | Strongly agree |
| --- | --- | --- | --- | --- | --- |
| feel I engaged with governance, strategy, policy and evaluation |  |  |  |  |  |
| advocated, led, engaged and informed continuous improvement of consumer engagement practice |  |  |  |  |  |
| provided strategic advice on consumer engagement |  |  |  |  |  |
| Provided strategic advice on consumer led education to the organisational leadership team |  |  |  |  |  |
| Provided strategic advice on research priorities/initiatives to the organisational leadership team |  |  |  |  |  |
| was/am involved in driving a program of consumer led consultation to build relationships and knowledge base in the diversity of experience and needs |  |  |  |  |  |
| had the opportunity to network, share information and engage in two-way communication and collaboration with researchers and other consumers |  |  |  |  |  |
| am aware of consumer activities research within the VCCC Alliance |  |  |  |  |  |
| Am aware of the role of consumer led research within the VCCC Alliance |  |  |  |  |  |
| am building relationships within the consumer led research group and VCCC Alliance project team |  |  |  |  |  |
| am exploring the definition, expectations, support and understanding of consumer led research |  |  |  |  |  |
| intend to apply the knowledge, skills and attitudes I have learned in this program in my role as a cancer consumer |  |  |  |  |  |
| felt comfortable about participating in this project given the support mechanisms in place. |  |  |  |  |  |

8. Has the VCCC Alliance Consumer-led Research Program changed your understanding of consumer-led research?
   c. Yes
   d. No
9. (Optional) Please describe how your understanding has/has not changed: (free text)

## 3.3 Education delivery, content and objectives

The next few questions are about the education/training that you received as part of this program. This education aimed to give you the knowledge and skills needed to be consumers leading research.

10. Did the instructor/facilitator present the course material in a way you could understand?
11. How useful was the education in helping you take part in the consumer-led research program?
    - Not at all useful
    - Slightly useful
    - Moderately useful
    - Very useful
    - Extremely useful
12. Was anything missing from the education?
    - Yes (please explain below)
    - No

Explain___________________________

13. If you have any other feedback or suggestions related to the education, please enter them here: _______________________

## 3.4 time invested and remuneration

14. How many hours approx. did you contribute to the consumer-led research program and related activities?
a. __ hours
15. Was this more/less/about the same as what you expected? (free text)
16. Was the money (remuneration) available for consumers enough?
a. Yes
b. No
17. Did you accept remuneration?
a. Yes
b. No
c. Prefer not to say
18. If you would like to add anything about the money (remuneration) available for consumers, please include it here (free text)

## 3.5 Experience working together

This section is about your experience working with your group of consumers during the consumer-led research program. Please respond to these questions thinking about the group of consumers in the program(s) you were involved in.

19. When working with others in the consumer-led research, how much did you feel: *(select one option per row)*

|  | 1. Never | 2. Rarely | 3. Sometimes | 4. Often | 5. Always |
| --- | --- | --- | --- | --- | --- |
| Comfortable asking questions and contributing ideas during meetings? |  |  |  |  |  |
| Included in conversations/decisions? |  |  |  |  |  |
| your contributions were valued? |  |  |  |  |  |
| you had the right level of knowledge/skills to contribute? |  |  |  |  |  |
| That the prioritization process was consumer-led? |  |  |  |  |  |
| the consumer group contained the diversity it needed? |  |  |  |  |  |
| You were supported to contact other team members including other consumers, facilitators and researchers? |  |  |  |  |  |

20. Is there anything that could be done differently to help you or other consumers work together? (free text)
21. If you have any further detail about working together, please enter it here: (free text)

## 3.6 Overall

22. How successful would you rate the VCCC Alliance Consumer-led Research Program? (*0-10 scale, where 0=Unsuccessful, 5=Neither successful or unsuccessful, 10=highly successful)*
23. (Optional) Provide more detail about the success of the program: (free text)
24. If you could change anything about the program, what would you change? (free text)
25. In your own words, what was the biggest challenge? (free text)
26. In your own words, what was the biggest success? (free text)
27. How likely are you to recommend the VCCC Alliance Consumer-led Research Program as a consumer to others? *(1-10 scale, where 1=not at all likely, 10=highly likely)*

28. Please explain why you chose the above rating: (free text)
29. Please share any further experiences or suggestions about how to improve consumer-led research in cancer: (free text)

**PART 1 – OTHER STAKEHOLDER QUESTIONNAIRE**

Project: Evaluation of VCCC Alliance Consumer-led Research Program

<redacted logo>

<Department and school name - redacted>

***Ethics project ID:*** <redacted>

**Thank you for your interest in participating in this evaluation project. For more information about this project, please see attachment here <PDF attachment of PLS>**

**We are interested in your thoughts on consumer-led research and how well this is working in the VCCC Alliance program(s) you are involved with.**

**The following questions are divided into two parts:**

Part 1 - About you (includes identifying information to measure demographics etc. Responses will be de-identified for analysis and reporting)

Part 2 - About your experience and views (stored separately to Part 1, no identifying information)

**Together, they will take 10-15 minutes to complete.**

10. I consent to participate in this project, the details of which have been explained to me, and I have been provided with a written plain language statement **<linked to document>** to keep.
11. I understand that the purpose of this research is to evaluate the VCCC Alliance consumer-led research framework and associated pilot projects.
12. I understand that my participation in this project is for research/evaluation purposes only.
13. I acknowledge that the possible effects of participating in this research project have been explained to my satisfaction.
14. In this project I will be required to complete a questionnaire that will take approximately 15 minutes.
15. I understand that my participation is voluntary and that I am free to withdraw from this project anytime without explanation or prejudice and to withdraw any unprocessed data that I have provided.
16. I understand that the data from this research will be stored at the <redacted> and will be destroyed 5 years after publication.
17. I have been informed that the confidentiality of the information I provide will be safeguarded subject to any legal requirements; my data will be password protected and accessible only by the named researchers.
18. I understand that by ticking the following box I am agreeing to take part in this research.

- **Yes, I agree to take part in this questionnaire**
- **No, I do not agree to take part in this questionnaire (this option will exit the survey)**

For more information, please contact the evaluation team:
Responsible Researcher: <redacted>

Tel: <redacted>Email: <redacted>

Additional Researchers:

<redacted> Email: <redacted>

<redacted> Email:  [<redacted>](mailto:Tessa.Saunders@unimelb.edu)

<redacted> Email: <redacted>

| **Evaluation of VCCC Alliance Consumer-led Research Part 1** | A A A |
| --- | --- |

**Page 2 of 3**

| Part 1 - About you | | |
| --- | --- | --- |
| **First name:** |  |  |
| **Last name:** |  |  |

1. What was your role in the VCCC Alliance Consumer-led Research Program?
   a. Consumer co-chair
   b. Consumer member
   c. Education development and delivery
   d. VCCC Alliance executive
   e. Researcher
   f. Educator/facilitator
   g. Program manager
   h. Other consumer (please describe)
   i. Other non-consumer (please describe)
2. How do you describe your gender?

*Gender refers to current gender, which may be different to sex recorded at birth and may be different to what is indicated on legal documents. Please select one box:*

a. Man or male
b. Woman or female
c. Non-binary
d. I use a different term (please specify)
e. Prefer not to answer

3. What was your age last birthday?_____________
4. In which country were you born?
   a. 11. Australia
   b. 12. England
   c. 13. New Zealand
   d. 14. India
   e. 15. Philippines
   f. 16. Vietnam
   g. 17. Italy
   h. 18. South Africa
   i. 19. Malaysia
   j. 20. Scotland
   k. 99. Other
5. Are you of Aboriginal origin, Torres Strait Islander origin, or both?
   a. Aboriginal
   b. Torres Strait Islander
   c. Both Aboriginal and Torres Strait Islander
   d. Neither Aboriginal or Torres Strait Islander
   e. Prefer not to answer
6. Which language do you mainly speak at home? Note, if more than one language, indicate the one that is spoken most often.
   a. English
   b. Mandarin
   c. Arabic
   d. Cantonese
   e. Vietnamese
   f. Italian
   g. Greek
   h. Hindi
   i. Spanish
   j. Punjabi
   k. Other (please specify)
7. What is the level of the highest qualification that you have completed?
   a. Postgraduate Degree
   b. Graduate Diploma/Graduate Certificate
   c. Bachelor Degree
   d. Advanced Diploma/Diploma
   e. Certificate III/IV
   f. Year 12 or equivalent
   g. Year 11
   h. Year 10
   i. Below Year 10/No educational attainment
8. What is your home postcode? (_ _ _ _)
9. Which of the following best describes your current employment status?
   a. Full-time employee
   b. Part-time employee
   c. Self-employed – not employing others
   d. Self-employed – employing others
   e. Unemployed – seeking part- or full-time work
   f. Not employed – not seeking employment
   g. Other
10. Have you ever worked with consumers before? If yes, please provide detail
    a. Yes
    b. No
11. Have you ever attended/completed any training/education in consumer involvement in research? If yes, please provide detail
    a. Yes
    b. No

**PART 2 – OTHER STAKEHOLDER QUESTIONNAIRE**

**The remaining questions ask about your experience and views of the consumer-led research program. All answers beyond this point will be anonymous. They will not be connected to your previous answers.**

## 3.7 Perceived purpose, role and responsibility of consumers in research

1. To make sure we ask you the right questions, were you a consumer or other stakeholder in this program?
   a. I was/am a consumer in this program
   b. I was/am another stakeholder in this program (this may include educators, facilitators, VCCC Alliance executive staff, program managers, researchers etc.)
2. Which program of work are/were you involved in?
   a. Program 2: Personalised cancer care
   b. Program 5: Value-based cancer care
   c. Both programs
3. In your opinion, what is the purpose of consumer involvement in research, in general? (free text)
4. In your own words, what was the main aim of the VCCC Alliance Consumer-led Research Program? (free text)
5. How often did you personally engage with the consumers in the consumer-led research program? *This may include sending/receiving emails, in person or telephone conversations or meetings*
   a. Weekly or more
   b. Monthly
   c. Infrequently (once or twice)
   d. Never
6. What was typically the purpose of this engagement? *(For example; providing support, delivering education, consultation…)* (free text)

### 3.7.1 Definition of consumer-led research

7. The following activities define consumer-led engagement. Please rate your level of agreement with the following statements in the context of this program: *(select one option for each row)*

|  | Strongly disagree | Disagree | Neither agree nor disagree | Agree | Strongly agree |
| --- | --- | --- | --- | --- | --- |
| Consumers engaged with governance, strategy, policy and evaluation |  |  |  |  |  |
| Consumers advocated, led, engaged and informed continuous improvement of consumer engagement practice |  |  |  |  |  |
| Consumers provided strategic advice on consumer engagement to the organisational leadership team |  |  |  |  |  |
| Consumers provided strategic advice on consumer led education to the organisational leadership team |  |  |  |  |  |
| Consumers provided strategic advice on research priorities/initiatives to the organisational leadership team |  |  |  |  |  |
| Consumers were involved in driving a program of consumer led consultation to build relationships and knowledge base in the diversity of experience and needs |  |  |  |  |  |
| Consumers had the opportunity to Network, share information and engage in two-way communication and collaboration |  |  |  |  |  |

8. Has the VCCC Alliance Consumer-led Research Program changed your understanding of consumer-led research?
   a. Yes
   b. No
9. (Optional) Please describe how your understanding has/has not changed: (free text)

#### 3.7.1.1 Experience working together

This section is about your experience working with your group of consumers during the consumer-led research program. Please respond to these questions thinking about the group of consumers in the program(s) you were involved in.

10. When working with others in the consumer-led research, how much did you feel: *(select one option per row)*

|  | 1. Never | 2. Rarely | 3. Sometimes | 4. Often | 5. Always |
| --- | --- | --- | --- | --- | --- |
| Comfortable asking questions and contributing ideas during meetings? |  |  |  |  |  |
| Included in conversations/decisions? |  |  |  |  |  |
| That your contributions were valued? |  |  |  |  |  |
| That you had the right level of knowledge/skills to contribute? |  |  |  |  |  |
| That consumers were comfortable asking questions and contributing ideas during meetings? |  |  |  |  |  |
| That consumers were included in conversations/decisions? |  |  |  |  |  |
| That consumer contributions were valued? |  |  |  |  |  |
| That consumers had the right level of knowledge/skills to contribute? |  |  |  |  |  |
| That the prioritization process was consumer-led? |  |  |  |  |  |
| That the consumer group contained the diversity it needed? |  |  |  |  |  |
| You were supported to contact other team members including other consumers, facilitators and researchers? |  |  |  |  |  |

11. If you have any further detail about working together, please enter it here: (free text)

#### 3.7.1.2 Overall

12. How successful would you rate the VCCC Alliance Consumer-led Research Program? (*0-10 scale, where 0=Unsuccessful, 5=Neither successful or unsuccessful, 10=highly successful)*
13. (Optional) Provide more detail about the success of the program: (free text)
14. If you could change anything about the program, what would you change? (free text)
15. In your own words, what was the biggest challenge? (free text)
16. In your own words, what was the biggest success? (free text)
17. How likely are you to recommend the VCCC Alliance Consumer-led Research Program or a similar program to colleagues/other organisations? *(1-10 scale, where 1=not at all likely, 10=highly likely)*

18. Please explain why you chose the above rating: (free text)
19. Please share any further experiences or suggestions about how to improve consumer-led research in cancer: (free text)

# 4 Interview guide

**Participants will be involved in program 2 and/or program 5. Program 2: Personalised cancer care (genomics etc). Program 5: Value-based cancer care.**

*Thank you <*participant*> for participating in this evaluation and taking the time to talk with me today, it is greatly appreciated.*

*My name is <*name> *I am from the <redacted>. I am part of the team evaluating the VCCC Alliance consumer led research. This evaluation is being conducted by the <redacted> and is funded by the VCCC Alliance.*

*You have been invited to participate because you have been involved with Consumer-led Research at the VCCC Alliance. We have invited consumers, educators, researchers, and program managers who were involved in the planning and delivery of the prioritisation activities and education modules.*

*I have a list of questions to guide us but would like this to be more like an informal conversation so feel free to jump in with any thoughts or ask me any questions at any time.*

*I’ll be asking for your views and experience, there are no right or wrong answers. It should take about 30 to 45 minutes and you can stop at any time if you change your mind.*

*As you know, I’m going to audio record this interview. I will do this in the zoom app and you will get a notification when it starts*

*While I’m recording, I ask that you try not to use any names or identifying information. So, instead of saying the name of a staff member or consumer you can just say my program facilitator or a consumer in my group. This will help keep you deidentified when we transcribe and analyse the interview.*

IF THEY REQUESTED THE TRANSCRIPT:

*I can see that you asked for a copy of the transcript after the interview. I will send it to you as soon as we have transcribed it and you can check it for accuracy and any identifying information. You will have two weeks from when I send it to you to respond with any changes or clarifications.*

*Do you have any questions before we start?*

IF NO;

*Ok, great, I received your consent form online, I would just like to confirm you would still like to participate in the evaluation today?*

IF YES

*I will start the recording now*

****start recording****

*To start, can you tell me a bit about your role in the consumer led research at VCCC Alliance?*

THEN MOVE TO CONSUMER/OTHER STAKEHOLDER QUESTIONS

For consumers:

| 1. | What are your overall thoughts on involving consumers in medical or health research?  Prompt: what level of involvement is most appropriate? |
| --- | --- |
| 2. | In your opinion, what are the core components of consumer-led research? |
| 3. | What do you see as the aim or purpose for the program? Do you feel these were met and why/why not |
| 4. | What advice would you give to new people in the program? |
| 5. | How comfortable do you feel working with other consumers and other stakeholders in the program? (what could be done to improve this?) |
| 6. | Did you feel that your contributions were valued by the researchers? |
| 7. | Do you feel you got more value/felt more valuable as a group rather than as individuals? (ask if they have had that previous experience as individual consumers in research) |
| 8. | What do you think are the biggest challenges for consumer-led research?  (prompts could lead to topics like diversity and inclusion, time, funding etc) |
| 9. | Did this program help you overcome any of these challenges?  Are there any other ways you can think of to overcome these challenges? |
| 10. | What part(s) of the program worked well for you? |
| 11. | What part of the program did not work effectively for you or your team? |
| 12. | Were the education and support sessions acceptable/feasible to you?  Why/why not?   a. Was there anything missing from the education that you feel would have been useful? |
| 13. | Do you feel the education components were useful for you as a consumer?  *If yes,*  Can you provide an example of this?  *If no,*  How do you think this could be improved? |
| 14. | Do you feel you had the right skills and knowledge to be involved in the program?  (prompt to ask if skills/knowledge was from education within the program) |
| 15. | What and how do you intend to apply what you have learned from the program? |
| 16. | Do you remember seeing/using the RACI matrix throughout the program?  *If no, briefly explain/show matrix*  What are your thoughts on the matrix and how it was used? |
| 17. | Can you think of anything that would help to improve the RACI matrix/definition of roles/responsibilities in the future? |
| 18. | What has your experience been like the last couple of months, after the prioritisation was completed and the research began?  Are there any different challenges compared to when you were getting set up? |
| 19. | Apart from in this evaluation, have you had the opportunity to provide any feedback to the VCCC Alliance team about the program?  has this been valued/acted upon etc? |
| 20. | Would you recommend consumer-led research at VCCC Alliance to others? |
|  | Why/why not? |
| 21. | Would you like to see this program continue? |
| 22. | Is there anything else you would like to discuss? |

For other stakeholders:

| 1. | What are your overall thoughts on involving consumers in medical or health research? |
| --- | --- |
| 2. | In your opinion, what are the core components of consumer-led research? |
| 3. | What do you see as the aim or purpose for the program? Do you feel these were met and why/why not |
| 4. | What changes have you had to make to your practice/way of working to more effectively involve consumers? |
| 5. | What advice would you give to other organisations setting up a similar program? |
| 6. | Do you feel that consumer involvement strengthened your research/added value? |
| 7. | How comfortable do you feel working with consumers and other stakeholders in the program? (what could be done to improve this?) |
| 8. | What do you think are the biggest challenges for consumer-led research?  (prompts could lead to topics like diversity and inclusion, time, funding etc) |
| 9. | Did this program help you overcome any of these challenges?  Are there any other ways you can think of to overcome these challenges? |
| 10. | What part(s) of the program worked well for you? |
| 11. | What part of the program did not work effectively for you or your team? |
| 12. | Do you remember seeing/using the RACI matrix throughout the program?  *If no, briefly explain/show matrix*  What are your thoughts on the matrix and how it was used? |
| 13. | Can you think of anything that would help to improve the RACI matrix/definition of roles/responsibilities in the future?  Are there any additional considerations for using this if the program was scaled up in the future? |
| 14. | Would you recommend consumer-led research at VCCC Alliance to others? |
|  | Why/why not? |
| 15. | Would you like to see this program continue? |
| 16. | Is there anything else you would like to discuss? |
